# Fabrication of X-ray absorption gratings by centrifugal deposition of bimodal tungsten particles in high aspect ratio silicon templates

**DOI:** 10.1038/s41598-022-08222-z

**Published:** 2022-03-30

**Authors:** Simon Pinzek, Alex Gustschin, Nikolai Gustschin, Manuel Viermetz, Franz Pfeiffer

**Affiliations:** 1 Chair of Biomedical Physics, Department of Physics, School of Natural Sciences, Technical University of Munich, 85748 Garching, Germany; 2 Munich Institute of Biomedical Engineering, Technical University of Munich, 85748 Garching, Germany; 3 Department of Diagnostic and Interventional Radiology, School of Medicine, Klinikum rechts der Isar, Technical University of Munich, 81675 Munich, Germany; 4 Institute for Advanced Study, Technical University of Munich, 85748 Garching, Germany

**Keywords:** X-rays, Optical materials and structures

## Abstract

Grating-based X-ray imaging employs high aspect ratio absorption gratings to generate contrast induced by attenuating, phase-shifting, and small-angle scattering properties of the imaged object. The fabrication of the absorption gratings remains a crucial challenge of the method on its pathway to clinical applications. We explore a simple and fast centrifugal tungsten particle deposition process into silicon-etched grating templates, which has decisive advantages over conventional methods. For that, we use a bimodal tungsten particle suspension which is introduced into a custom designed grating holder and centrifuged at over 1000×*g*. Gratings with 45 µm period, 450 µm depth, and 170 mm × 38 mm active area are successfully processed reaching a homogeneous absorber filling. The effective absorbing tungsten thickness in the trenches is 207 µm resulting in a filling ratio of 46.6% compared to a voidless filling. The grating was tested in a Talbot–Lau interferometer designed for clinical X-ray dark-field computed tomography, where visibilities up to 33.6% at 60 kV were achieved.

## Introduction

In the last two decades, X-ray grating interferometry (XGI) was used to develop imaging systems that gain additional information to conventional X-ray radiography. Two new modalities sensitive to phase-shifting and small-angle scattering properties of the imaged objects were introduced and are referred to as differential phase and dark-field imaging due to similarities with the respective techniques known from light microscopy^1–5^. Since then many potential applications including promising preclinical studies have been reported^6–11^. Recently, the method was successfully adapted for in vivo human thorax imaging exploring its first diagnostic abilities^12^. A common implementation of such an imaging system uses a phase grating G$_1$ to induce an intensity modulation at so-called fractional Talbot-distances^13^ if the radiation is sufficiently coherent. To create such a modulation with conventional, incoherent X-ray sources an absorption grating G$_0$ can be used employing the Lau effect^3^. Since this modulation is usually too small to be directly resolved by conventional X-ray detectors an additional absorbing grating G$_2$ with a similar period like the modulation is employed to create a Moiré fringe pattern. Using either phase stepping or scanning the change of the intensity modulation induced by a sample can be retrieved in each pixel.

The image quality of such a system depends strongly on the grating quality. Especially the absorption gratings pose a problem since they require high aspect ratio (ratio of width to height of the structures) structures in the µm-range that periodically absorb the X-ray radiation. One suitable method to fabricate such absorption gratings is the so-called direct deep X-ray LIGA-process (germ. Lithographie, Galvanik, Abformung). At first, a thick resist layer is coated on a conductive substrate and exposed to a masked X-ray beam (deep X-ray lithography). Then, the structure is chemically developed and filled with gold (Au) by electroplating^14–17^. To achieve high aspect ratios the steep resist structures have to be connected with supporting structures (‘bridges’) which reduce the grating performance. The X-ray lithography step is usually performed at synchrotron facilities since it requires a certain degree of beam coherence, adaptable energy, and bandwidth as well as high intensity. This limits the availability of the method for development and high volume production.

Other methods for the fabrication of absorption gratings are based on different silicon etching techniques like anisotropic wet etching^18,19^, plasma etching^20–23^, photo-assisted electrochemical etching^24,25^, or metal-assisted chemical etching^20,26,27^. Here, the deep trenches are etched into the silicon substrate and need to be filled with a strongly absorbing material. If Au plating is applied certain additional processes need to be performed to deposit a suitable conductive layer. The challenge is to prevent a conformal process that tends to close the trenches from the top and leaves voids in the trenches and an unwanted Au layer on top of the grating structure. For this purpose, a selective plating contact deposition on the bottoms of the trenches was demonstrated^18^. Alternatively, an adapted grating structure can be covered by atomic layer deposition and conformably plated where the material deposited on the sidewalls is used as the absorbing part^18^. Grating templates created by metal-assisted chemical etching already have a conductive seed layer that can be used as a plating base^26,27^. It was also proposed to make the substrate conductive by doping the silicon with Au and subsequent passivation of the grating sidewalls and the surface by thermal oxidation^28^. Similarly, using conductive substrates and creating gratings with a slight bottom tapering could fill the structure conformally avoiding voids^29^. Furthermore, there are approaches to optimize the chemistry and kinetics of the plating process to improve the filling of the trenches^30,31^. The advantage of all those methods is that the grating template can be produced with widely available silicon processing technology unlike with the LIGA process. However, all of these methods still use Au as an absorbing matrix which comes at a high material cost and an elaborate, error-prone electroplating process.

As an alternative to electroplating, the deposition of absorbing material in a molten state has been proposed. A micro-casting approach in a pressure-controlled chamber has been demonstrated with bismuth^32^. Others suggested an imprint process with metallic glass containing highly absorbing elements such as palladium^33^ and platinum^34^. Furthermore, hot embossing with low melting point alloys of lead and Au has been demonstrated^20^. This approach is limited by the melting point of the absorber and its thermal properties as the silicon grating structure has to remain intact upon pressure application and cooling.

Another alternative is to deposit the absorbent filling into the trenches in the shape of small particles. Empty cavities between the particles can still be accepted if it is compensated by an increased trench depth. Such a filling was first demonstrated successfully for neutron absorption gratings with a period of 774 µm and trench depth of 500 µm using gadolinium oxysulfide particles^35^. It has been optimized down to periods as small as 13.3 µm and performed well even in the thermal neutron range^36^. For X-ray absorption gratings Lei et al.^37^ filled gratings of 42 µm period, 31.5 µm trench width, and 150 µm height with tungsten (W) nanoparticles. The method was scaled to an 8inch (200 mm) silicon wafer and the filling ratio in the trenches was estimated to be 33%^38^. In a cascaded Talbot–Lau interferometer operated at 40 kV tube voltage, the grating performed with up to 10% visibility^38^.

Hojo et al.^39^ aimed to increase the filling ratio by forcing 1.5 µm Au particles into the trenches using an ultra-centrifuge. They created a custom holder which allowed them to fill a small grating piece with a centrifugal acceleration of 162,000×*g*. The period was 30 µm, the trench width 20 µm, the height 67 µm, and a filling ratio of 65% was estimated^39^. As these examples show, the aspect ratios of particle-filled X-ray absorption gratings have not exceeded 1:5 yet. Furthermore, the demonstrated gratings had a duty cycle (DC, ratio of etched/absorbing area to period) significantly larger than 0.5 which makes them unfavorable as analyzer gratings for dose-sensitive applications.

In this paper, we present the fabrication of an 170 mm × 38 mm sized X-ray absorption grating with a period of 45 µm and a trench depth of 450 µm (aspect ratio $\approx$ 1:20). To increase the particle filling ratio, a bimodal tungsten particle mixture is used. The deposition is carried out in a custom designed centrifugation container for large grating substrates. After deposition, we characterize the grating by scanning electron microscopy (SEM), X-ray microscopy (XRM), and angular X-ray transmission analysis (AXT)^40^. We compare the W particle-based grating with a LIGA-fabricated grating in a compact Talbot–Lau imaging setup resembling a possible computed tomography (CT) scanner geometry.

## Materials and methods

### Fabrication of particle-based absorption gratings

Filling a volume with particles and optimizing its effective density is subject of numerous theoretical and empirical studies and of interest for many applications such as material sciences, metallurgy, or logistics. It is known that a random arrangement of equal spheres fills the volume to about 55% (loose random packing), which can be increased to 64% by external interaction like shaking or applying pressure (random close packing)^41^. A further increase can be reached by using different particle sizes to fill the space between large particles with smaller ones. When a bimodal mixture is used (two different particle sizes), the ratio of their radii and their volume fractions are of importance^42^. The bigger the difference in the radii, the better the cavities between the larger particles can be filled. However, there are several unfavorable effects like walling, loosening, and wedging that decrease the theoretical density predicted by simple models^42^. Usually, optimal packing densities are achieved when the volume fraction of coarse particles is in the range of 0.65–0.8 and the radii differ in one order of magnitude or more^42^. In one such study, the packing has been increased from 53% to 59% by using a bimodal mixture^42^.

Filling high aspect ratio structures introduces additional effects like empty cavities at the trench walls especially when the coarse particles are in the same size range as the trench opening. Due to reduced mobility in the trenches, there is more potential to get stuck and prevent other particles to rearrange and find favorable positions. Furthermore, commercially available particles are usually not spherical as assumed in the models and also have a significant dispersity in size. Besides, physical and chemical interactions become more relevant with smaller particle sizes and can affect such micromorphologies significantly. Keeping all these factors in mind it is reasonable to explore what particle sizes are most suitable for grating deposition and if a bimodal mixture can increase the filling density.

**Figure 1:**
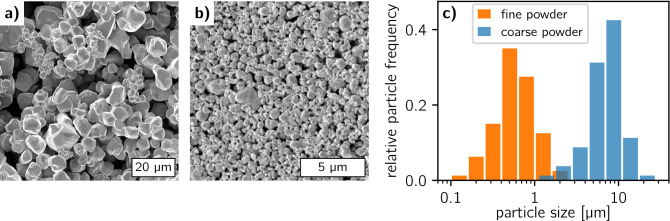
SEM images of (**a**) the coarse and (**b**) the fine tungsten (W) particle powder. (**c**) Particle size distribution for both particle powders determined from 40 representative particles for each powder. A mean particle size of $7.7 \pm 3.4$ µm and $0.66 \pm 0.35$ µm is determined for the coarse and the fine W particle powder, respectively.

As absorbing material, we chose W as it has a high density comparable to Au and is commercially available in different particle sizes at negligible cost compared to Au. We obtained a coarse powder (W, 12 µm, 99.9%) and a fine powder (W, < 1 µm, 99.95%) from Sigma Aldrich (Sigma Aldrich, St. Luis, USA) and prepared some samples to determine the particle size distribution. For that, we measured the size of 40 representative particles from both powders in SEM images. Figure 1a,b show SEM images of the coarse and fine powders and c) provides histograms of the measured particles size distributions. For the coarse powder, the particle size was estimated to be 7.7 ± 3.4 µm and for the fine powder, it was $0.66 \pm 0.35$ µm—about one order of magnitude smaller. The bimodal mixture was composed by weight in a ratio of 2:1 (coarse powder : fine powder), resulting in a coarse particle weight fraction of 0.66, which is in the optimized range as discussed before.

As a carrier of the particles, we dissolved Polyvinylpyrrolidone (PVP, 10,000g mol$^{-1}$, Sigma Aldrich, St. Luis, USA) in ethanol in a concentration of 1 mg mL$^{-1}$. The PVP polymer serves as a binder fixing the particles in the trenches after evaporation of the ethanol. Both powders and their bimodal mixture were suspended in the carrier with a particle concentration of 0.45 g mL$^{-1}$, resulting in a W/PVP-weight ratio of 450:1. The suspensions were magnetically stirred at 300 rotations per minute to prevent particle sedimentation and achieve good mixing.

**Figure 2:**
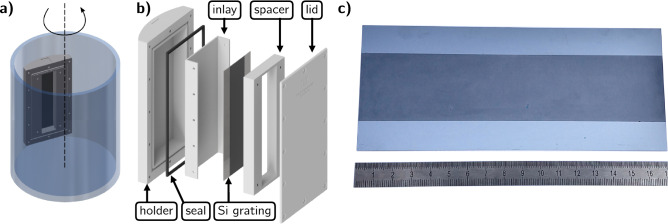
(**a**) Illustration of the centrifuge drum with the grating insert. (**b**) The insert consists of a holder with a cavity for the grating inlay and the particle suspension. The inlay helps to remove the grating after centrifugation and the spacer confines the volume in the particle reservoir to the grating area. The lid and the seal close the vessel and prevent leakage. The silicon (Si) grating template is placed between inlay and spacer. (**c**) Photograph of the fabricated 170 mm × 38 mm sized grating next to a cm ruler.

To develop the deposition process, we started with small grating pieces (10 mm × 10 mm) with a period of 36 µm, a depth of 170 µm, and a DC of 0.60 fabricated by deep reactive ion etching (DRIE). For the deposition, we used a spin dryer centrifuge (oneConcept WS-3500) with a drum of 240 mm diameter. A custom sample container that can be magnetically attached inside the rotating drum and carries the grating piece and the particle suspension was designed and 3D printed. Figure 2a illustrates the arrangement where the centrifugal force is acting perpendicularly onto the grating substrate upon drum rotation. Three samples were prepared to compare the filling of the coarse and fine powder individually, as well as their bimodal mixture. Each grating piece was inserted into one sample container and wetted with ethanol to prevent air bubbles after the application of the particle suspension. We found a proper wetting by ethanol even in the deep silicon trenches without any additional surface treatment after DRIE. Subsequently, the suspensions were dripped onto the grating structure with a pipette until the container reservoir was filled. Then every vessel was sealed with the lid, placed into the centrifuge drum, and counterbalanced with an object of comparable dimensions and weight to prevent instabilities and vibrations upon drum rotation. The centrifugation was carried out at 2800 rotations per minute, resulting in a centrifugal acceleration of 1050×*g* for 5 min. Subsequently, each container was flipped and centrifuged a second time for 5 min as the particle concentration tends to increase at the bottom of the vessel due to sedimentation resulting in uneven particle distribution. After that, each vessel was opened and placed in a fume hood at ambient conditions to allow the ethanol to evaporate and the sample surface to dry overnight. The grating pieces were carefully extracted from the vessels and investigated under a light microscope. Their surfaces were fully covered with protruding particles, which were removed by hand using a razor blade or a scalpel under the microscope. It is essential to ablate the particle layer moving the blade parallel to the grating lines to avoid any damage to the grating structure.

After promising results with small grating pieces, we developed the method further to scale the process in area and fill structures with higher aspect ratios. The objective was to fabricate a grating for a compact inverse Talbot–Lau interferometer system recently described for CT application^43^. For that, we acquired a custom designed 170 mm × 38 mm sized grating fabricated by DRIE on an 8 inch wafer from Fraunhofer IPMS (Dresden, Germany) with a period of 45 µm, a DC of 0.54 (which is equivalent to a trench width of 24.3 µm), and a depth of 450 µm. The grating lines are orientated parallel to the short edge of the grating template and the etched trenches are surrounded by 16 mm unetched silicon substrate (see Fig. 2c). A custom centrifuge container (Fig. 2b) was designed and 3D printed consisting of a holder with an inner cavity containing an inlay for easier grating extraction after centrifugation. A spacer reduces the reservoir for the particle suspension to the volume above the active grating area and a lid with an EPDM seal closes the vessel. The silicon grating template was inserted into the container and wetted with ethanol as previously described. Then, the reservoir was filled with the bimodal particle suspension, sealed, and centrifuged two times for 5 min after a flip in-between applying the same parameters as previously described. After that, the vessel was opened and inspected. The suspension cleared up as the majority of the particles were deposited into the grating trenches, which were not yet filled, as observable under a microscope. The remaining fluid was suctioned with a pipet and the reservoir was refilled with some more bimodal particle suspension for a second centrifugation applying the same protocol. After removing the residual liquid, the grating was covered one more time with the fine particle suspension to fill the remaining voids between the large particles on the surface of the grating. Afterwards, the substrate was allowed to dry overnight as previously described. Despite the large grating area, the residual particle layer on the surface could be cleanly removed mechanically as previously described in about 1 h. To estimate the filling density, we weighted the substrate before particle deposition and after surface cleaning finding a weight increase of 13.9 g. A photograph of the processed grating is shown in Fig. 2c. Additionally to the large area grating, a smaller edge piece that was fabricated on the same wafer with the same process parameters was filled with the bimodal particle mixture with the same protocol. This piece was cleaved to investigate the grating profile and its filling via SEM.

### Grating characterization methods

Grating characterization was performed with different complementary techniques such as SEM, XRM, AXT and a visibility analysis in a compact Talbot–Lau interferometer. While surface analysis techniques such as SEM provide a high resolution and allow to get a good impression of the particle filling in the trenches of cleaved grating pieces, X-ray techniques can probe the inner grating structures non-destructively. In many cases, XRM can even resolve individual grating lines and provide good estimates on period, DC, absorbing power, and inclination of the lamellae. However, it is unsuitable for large area characterization due to its small field of view and long data acquisition process. AXT allows probing most of the grating parameters on a large grating area in a reasonable time frame with a sub-mm resolution^40^.

**Figure 3:**
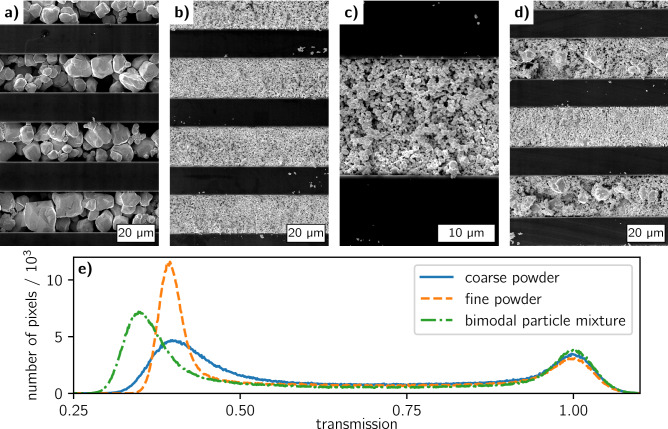
SEM images of the 10 mm × 10 mm sized absorption gratings with 36 µm period filled with (**a**) the coarse W particle powder, (**b**,**c**) the fine W particle powder, and (**d**) the bimodal mixture of coarse and fine (ratio 2:1) W particle powders. (**e**) Histograms of high-resolution X-ray transmission images of the three gratings.

For a first impression of the particle shape, size, and morphology in the grating trenches we acquired SEM images of the sample pieces with a JEOL JSM-6060LV (JEOL, Freising, Germany) SEM. Figure 3a–d show some surface images of the 10 mm × 10 mm grating pieces with different particle fillings. To obtain in-depth information and compare the three particle fillings in terms of their attenuation performance, high-resolution radiographs were acquired with an Xradia 500 Versa X-ray microscope at 100 kV and 70 µA tube parameters and an exposure time of 120 s. By a combination of optical and geometrical magnification, the effective pixel size was 1.04 µm which allowed to resolve the structure well. To compare the three gratings, the radiographs were normalized to their maximum transmission and histograms of the pixel transmission values were extracted (Fig. 3e).

**Figure 4:**
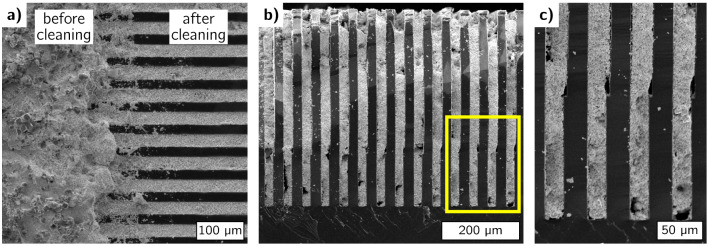
SEM images of the small grating piece filled with the bimodal W particle mixture. (**a**) The grating surface is shown after the particle deposition (left) and after removing the protruding particles from the surface (right). (**b**) A cross-sectional view of the grating after it has been cleaved, and (**c**) a magnified view of the region in the yellow rectangle.

To investigate how the filling could be performed with higher aspect ratios, a small grating piece with the same parameters as the large grating was examined by SEM. Figure 4a shows the surface where the residual particle layer was not removed on the left side in contrast to the right side, which has been cleaned with a doctor blade. After surface cleaning, this small grating piece was cleaved to obtain a cross-sectional view of the particle filling. Figure 4b shows the grating profile with the filled trenches and a magnified view of the yellow rectangle is provided in c.

**Figure 5:**
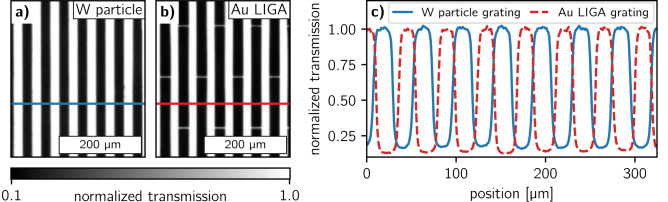
Normalized high-resolution transmission images of (**a**) the 170 mm × 38 mm sized grating filled with the bimodal tungsten (W) particle mixture and (**b**) of a state-of-the-art gold (Au) LIGA grating for comparison. (**c**) Line plots of the normalized transmission for both gratings along the colored lines in (**a**) and (**b**).

The large grating was directly compared with a state-of-the-art Au LIGA grating (microworks GmbH, Karlsruhe, Germany) with 280 µm absorber height, 45 µm period, and a DC of 0.54 fabricated on a graphite substrate. XRM images of both samples were acquired with the aforementioned system at 80 kV and 5.5 W with an exposure time of 45 s. The XRM system in this configuration had a resolution of 3.8 µm (full width at half maximum of the line spread function) as determined from an error function fit of an absorbing edge. Since both gratings have different substrates and thus slightly different transmittance on the transparent lines, they were normalized to their maximum values. Transmission micrographs of both gratings are provided in Fig. 5a, b and a line plot of the normalized transmission is plotted in c.

**Figure 6:**
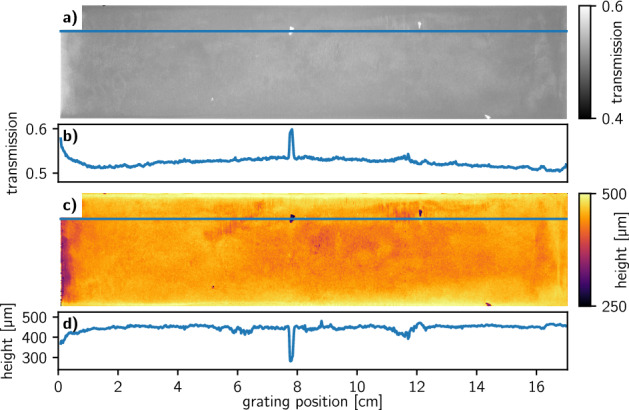
Angular X-ray transmission analysis results for the 170 mm × 38 mm sized grating filled with the bimodal W particle mixture. (**a**) Transmission image that has been virtually recorded with a parallel beam geometry and (**c**) geometrical height image. Corresponding line plots along the blue lines are given in (**b**) and (**d**).

To investigate the grating quality over the entire surface of the large grating, an AXT analysis^40^ was performed using a phoenix$\mid$X-ray v$\mid$tome$\mid$x (General Electrics) microCT system. The tube was operated at 80 kV tube voltage and 75 µA tube current. 800 transmission images with an exposure time of 6 s were acquired while the grating was rotated over an angle range of ± 40°. This allowed to reconstruct the grating transmission (see Fig. 6a with a respective line plot in b) over the entire area without shadowing artifacts that typically arise with cone-beam systems when the gratings are not bent to the respective radius. The transmission image is sensitive to DC variations, poorly filled trenches, or defects in the grating structure^40^. In addition, the angular data allows to reconstruct a spatial height map of the grating, which is provided in Fig. 6c with a respective line plot in d.

**Table 1:** Experimental parameters and results of the Talbot–Lau interferometer used for visibility comparison and imaging. The ± sign indicates the standard deviation of the visibility.

parameter	W particle grating	Au LIGA grating
X-ray source	XWT-160-CT, X-rayWorX	
X-ray filter	3 mm Al + 0.5 mm Mo	
Source-G$_0$ distance/bending radius	$\approx$ 100 mm	
G$_0$ (period/DC/height)	4.8 µm/0.5–0.6/280 µm Au	
G$_1$-source distance/bending radius	$\approx$ 200 mm	
G$_1$ (period/height)	4.34 µm/18.5 µm Au triangular	
G$_2$-source distance/bending radius	$\approx$ 1000 mm	
G$_2$ (period/DC/height)	45.0 µm/0.54/450 µm Effective height 207 µm W	45.0 µm/0.54/280 µm
Phase stepping	G$_2$,45.0 µm range, 11 steps	
Source-sample distance	$\approx$ 580 mm	
Detector	XRD 4343CT (Varex)—2s exposure time	
Visibility 60 kV	(33.6 ± 1.4)%	(32.4 ± 1.1)%
Visibility 70 kV	(26.0 ± 1.3)%	(28.7 ± 1.3)%
Visibility 80 kV	(21.1 ± 1.2)%	(23.5 ± 1.3)%

**Figure 7:**
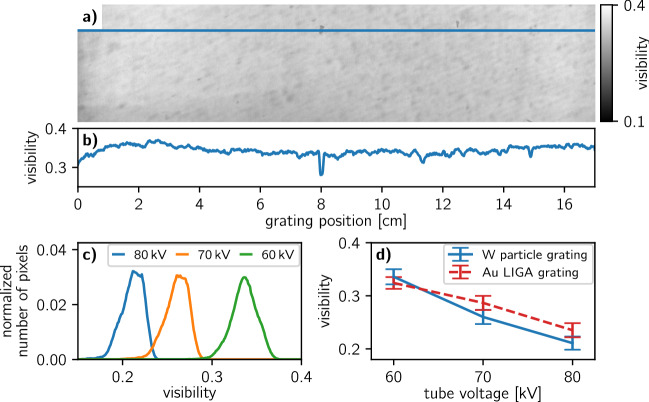
Characterization of the fabricated W particle grating in an X-ray grating interferometer. (**a**) Visibility in a Talbot–Lau interferometer for X-ray dark-field computed tomography, in which the large W particle grating was employed as G$_2$, at 60 kV tube voltage and (**b**) the corresponding line plot along the blue line in (**a**). (**c**) Visibility distribution of the interferometer for 80 kV, 70 kV, and 60 kV tube voltage. (**d**) Comparison of the visibilities using the here presented W particle grating and a state-of-the-art Au LIGA grating as analyzer grating. The error bars indicate the standard deviation of the visibilities.

**Figure 8:**
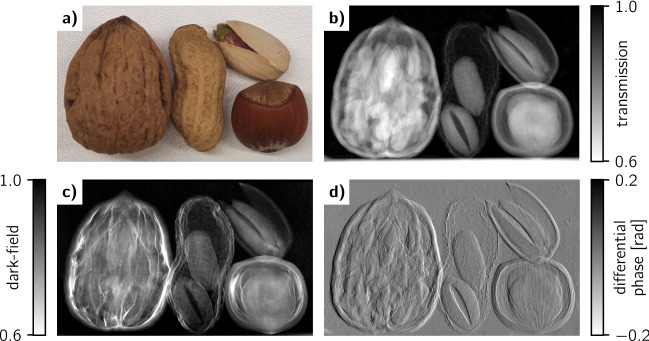
Imaging example using the fabricated W particle grating as analyzer grating in an X-ray grating interferometer. (**a**) Photograph of the imaged nuts, namely a walnut, a peanut, a pistachio, and a hazelnut. (**b**) Transmission image, (**c**) dark-field image, and (**d**) differential phase image of the four nuts acquired at 60 kV tube voltage.

As the final characterization step for the fabricated grating, we tested it in a compact, inverse Talbot–Lau interferometer geometry designed for clinical X-ray dark-field CT. Such setups allow to use analyzer gratings with relatively large periods and therefore reduce the requirements for the aspect ratio^44^. At the same time, the geometry requires relatively small G$_0$ and G$_1$ gratings with high aspect ratios. The grating parameters and the used hardware are given in Table 1 and more information on the design can be found in a recently published article^43^. The described geometry of the clinical X-ray dark-field CT was replicated in a laboratory setup to characterize the grating at a high resolution. The W particle grating was also compared with a Au LIGA grating (microworks GmbH, Karlsruhe, Germany) at 60 kV, 70 kV and 80 kV tube voltage at 40 W tube power. The X-ray source was filtered with 3 mm of aluminium and 0.5 mm molybdenum to obtain hard X-ray spectra similar to medical CT applications. Figure 7a shows the visibility map with a respective line plot in b acquired at 60 kV. Histograms of the achieved visibilities using the W particle grating at different tube voltages are shown in Fig. 7c. A comparison of average visibilities and respective standard deviations for the W particle and the Au LIGA grating is shown in Fig. 7d. To demonstrate the imaging capabilities of the system, we composed a sample of different nuts shown in Fig. 8a. We performed a phase stepping with the parameters given in Table 1 at 60 kV tube voltage and processed the image with an expectation-maximization-assisted sinusoidal least square fitting. Since the sample did not fit into the field of view in the vertical direction, the sample was stepped in height acquiring 3 images with overlap and stitched together. Figure 8b shows the transmission image, c the dark-field image, and d the differential phase image.

## Results and discussion

### Comparison of different W particle fillings

The surface SEM images of the three grating pieces (Fig. 3a–d) show that the transmitting silicon lines are mostly clear and only a few small residual particles remain on the area that is supposed to be X-ray transparent. The coarse particle filling (Fig. 3a) reveals voids especially close to the trench walls and it is to be expected that their abundance increases with depth as their mobility is constrained. The fine particle filling (Fig. 3b) gives a more uniform impression, however, a closer look at increased magnification (Fig. 3c) also reveals a high porosity. An optimization of the particle carrier solution using different solvents and binders might decrease the voids between the particles resulting in a better filling. The bimodal filling (Fig. 3d) is difficult to be interpreted, as the distribution of the coarser particles in the trenches is not well recognizable due to the covering of the fine particles. A more quantitative and reliable insight probing the filling also in depth is provided by the XRM data. The histograms of the pixel transmission values (Fig. 3e) show that the coarse and the fine powder filling have comparable transmission (0.4) at the absorbing areas. However, in the case of the coarser filling, there is a broader distribution of the transmission values indicating that there are areas with significantly higher or lower filling density. The latter might be the case at the trench edges as there are more voids in the vicinity of the trench walls. The bimodal particle filling has significantly better absorption properties as its average transmission is at 0.35 and it clearly reduces the fraction of transmission values in the range of 0.4–0.5. This suggests that the finer particles in the bimodal mixture fill cavities close the edges helping to achieve a sharper grating transmission profile. Therefore, we decided to use the bimodal particle mixture for deposition into the large area grating.

### Characterization of the large area W particle grating

The W particle mixture of the 45 µm period grating as seen from the SEM images (Fig. 4) covers the trenches throughout the entire depth of 450 µm. Several larger bubble-like voids are visible, however, it is difficult to tell how much of them were created due to the breaking of the substrate to visualize the profile. Measuring the trench opening at different depths of the grating, we estimated an average DC of 0.54. XRM measurements of the grating (Fig. 5a) show a well-defined linear pattern with sharp edges comparable to the Au LIGA-fabricated grating (Fig. 5b). Line plots of the transmission profiles (Fig. 5c) show 16% residual transmittance for the W particle grating and 13% transmission for the Au LIGA grating on the absorbing areas. Slightly better absorption performance of the Au LIGA grating is expected, as its attenuating lamellae have a significantly higher effective thickness. Unlike the silicon-based W particle grating, the Au LIGA grating has X-ray transparent resist stabilization structures (‘bridges’) between the transmitting lines as seen in Fig. 5b. Those structures reduce the performance of the absorption gratings especially at smaller periods when the fraction of bridge-related transparent area becomes relatively high. Using silicon-based grating templates usually allows avoiding such unfavorable structures.

After promising SEM and XRM findings, a large area characterization of the grating structure is necessary to confirm that the presented deposition process can be conducted homogeneously over the entire active area without damaging the fine grating structure. The AXT analysis shows that the grating transmission (Fig. 6a) is mostly uniform between 0.5 and 0.55 over the entire grating area. Only a slight increase is recognizable at the left edge and some local defects with an increased transmission are present as depicted by the line plot in Fig. 6b. At the same time, the heightmap (Fig. 6c) shows a reduced height on the same spots. The line plot of the height (Fig. 6d) shows a reduction to slightly below 300 µm at the defect in the middle of the grating and a reduction to 380 µm on the left grating edge. Note here that the AXT analysis provides the filled height of the grating structure^40^ and not the effective height, which denotes the thickness of the absorbing material. Hence, the spot-like defects are not voids in the trenches but deficiencies in the etched grating template and can therefore not be filled. The area with a reduced transmission on the left might be caused by insufficient filling during centrifugation. As the grating is placed into the insert with the particle reservoir, the bottom area might be blocked by larger particles which sediment much faster and compromise the penetration of smaller particles into the trenches. Overall, an average height of 450 µm particle filling in the trenches is observed and coincided with the design values.

Considering the local height distribution for each pixel, the DC of 0.54, and a weight increase after particle deposition (13.9 g) an average filling density of 46.6% can be estimated. This is significantly higher than a recent work with a slightly larger trench width by Lei et al.^38^ estimating 33% filling and lower than the ultracentrifugation approach reporting 64% by Hojo et al.^39^ with a slightly smaller trench width compared to our grating. In our case, however, the aspect ratio was several times higher than previous works and resulted in an average effective W filling of 207 µm. Furthermore, we showed that a centrifugation approach—albeit at a much lower rotation speed—can be also realized with much larger grating areas compared to^39^.

The final characterization performed in a Talbot–Lau interferometer yields a homogeneous visibility map (Fig. 7a) and an average of 33.6% at 60 kV tube voltage. The central defect discussed before degrades the visibility to about 28% as denoted by the line plot in Fig. 7b. Some further spots are visible in the visibility map, which are not apparent in the transmission image from the AXT analysis (Fig. 6a). Those are grating defects in the $G_0$ and $G_1$ grating since they were also present with the Au LIGA grating. Histograms of the visibility maps at increasing tube voltages (Fig. 7c) show a visibility decrease. This is expectable as the gratings become more transparent with harder X-rays. The interferometer-based visibility comparison with the Au LIGA grating shown in Fig. 7d reveals a comparable visibility performance at 60 kV while at higher voltages the W particle grating performs slightly worse than the Au LIGA grating. At higher energies the significantly higher absorption thickness of the Au bars (280 µm) and its stronger attenuation coefficient compared to W absorb better and explain this difference. Overall, the visibility performance of both gratings differs only in the range of a few percent. An imaging example (Fig. 8) using the fabricated W particle grating at 60 kV tube voltage yields excellent, artifact-free images confirming their applicability for hard X-ray phase-contrast and dark-field imaging.

## Conclusion

In this paper, we presented a method to deposit W micro-particles into high aspect ratio grating structures by centrifugation. That allows using conventional micropatterning and etching technologies such as UV-lithography and DRIE (available up to 300 mm wafers) to fabricate grating templates without the need for further special processing steps like seed layer deposition and electroplating. The particle deposition process itself does not require controlled environment and sophisticated equipment. Furthermore, the method allows using significantly cheaper alternatives to Au as absorber materials. W is commercially available in a high variety of powders due to its wide applications in metallurgy and has a similar density to Au. Its lower K-absorption edge at 69.5 keV might also be considered as an advantage compared to Au, however, will only play a considerable role for systems with significantly higher design energy than the system presented here. Furthermore, we found that a bimodal particle mixture improves the filling density compared to a coarse or fine powder only.

We successfully fabricated a 170 mm × 38 mm sized grating using the developed process and achieved a filling ratio of 46.6% and an effective W height of 207 µm. The latter clearly exceeds recently reported studies^37–39^ and allows the grating to be used for hard X-ray applications. In a direct comparison with a LIGA-fabricated grating conducted in an inverse, compact Talbot–Lau setup similar visibilities were reached.

The main bottleneck for a clinical application of X-ray phase-contrast and dark-field imaging remains the fabrication of the required absorption gratings. For complete coverage of the field of view of e.g. a thorax radiography system or a CT scanner, the $G_2$ grating poses the most effort and cost. Hence, research towards reliable and cost-effective fabrication techniques such as the one presented here should gain priority. For inverse Talbot–Lau systems source gratings with very small periods and extremely high aspect ratios are required^44^. Thus, advanced technologies like LIGA with their dense trench filling by electroplating Au are indispensable for these gratings. Furthermore, flexible substrates like graphite are compatible with LIGA and allow to create gratings that can be bent to radii down to 100 mm which is necessary for compact systems. However, for analyzer gratings with their large active area material cost become more important. At the same time, requirements regarding period and aspect ratio are more relaxed in inverse Talbot–Lau systems. For these gratings, the here presented low-cost fabrication method based on the deposition of W particles is a promising alternative. To increase design flexibility, the next goal becomes to downscale the described particle deposition process to a period in the range of 10 µm to 20 µm while maintaining effective W absorbing thicknesses above 200 µm. Fabrication of suitable silicon templates with even higher aspect ratios requires a precise profile control in DRIE to maintain a constant duty cycle. Furthermore, grating lamellae with smaller periods might stick to each other due to capillary forces introduced by the fluids or break due to the centrifugal forces. To address these stability issues the grating lines can be connected by supporting bridge structures similar to LIGA-based gratings. A further challenge might be a reduced particle filling density when smaller particles are used. To cope with that the particle suspension has to be optimized with a focus on surface-related inter-particle interactions. Reaching good absorbing performance with periods in the sub-20 µm length scale would allow producing cost-effective and sufficiently sensitive systems for e.g. thorax dark-field radiography, which recently demonstrated its first clinical application^12^.
